# Supplementary material for: Dose Effect of Polyethylene Microplastics Derived from Commercial Resins on Soil Properties, Bacterial Communities, and Enzymatic Activity
Source: Microorganisms. 2024 Aug 29;12(9):1790. doi: 10.3390/microorganisms12091790 (PMC11434124; doi:10.3390/microorganisms12091790)
Supplement: Supplementary file 1 [file microorganisms-12-01790-s001.zip › microorganisms-3157146-supplementary.pdf]

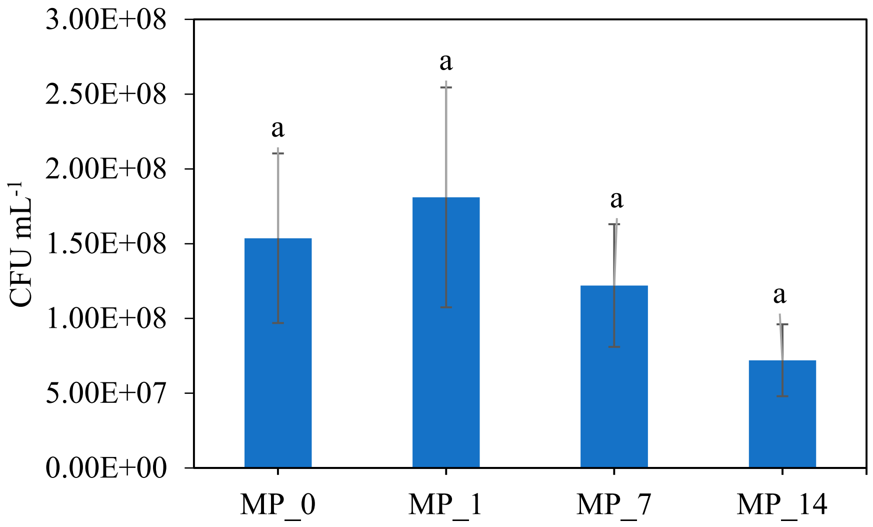

Figure S1. CFU at different treatments of PE MPs in nutrient rich agar plate

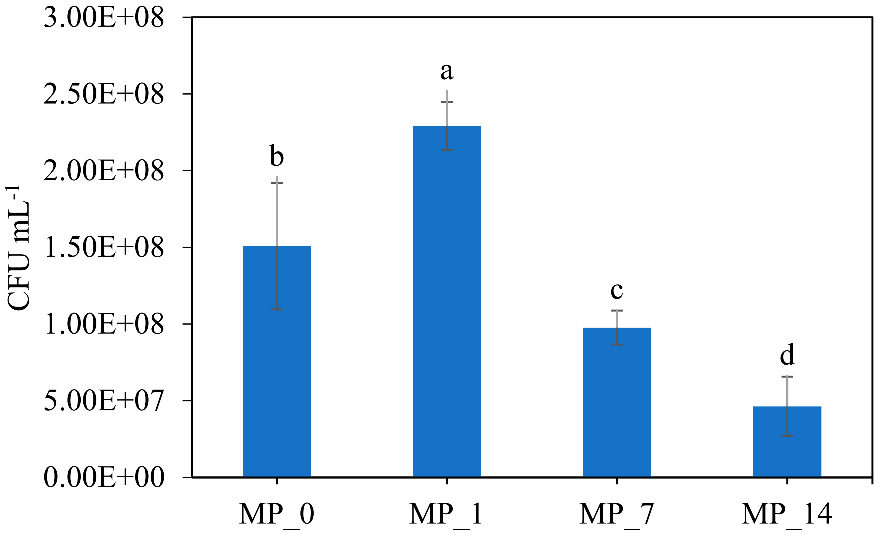

Figure S2. CFU at different treatments of PE MPs in nutrient poor agar plates

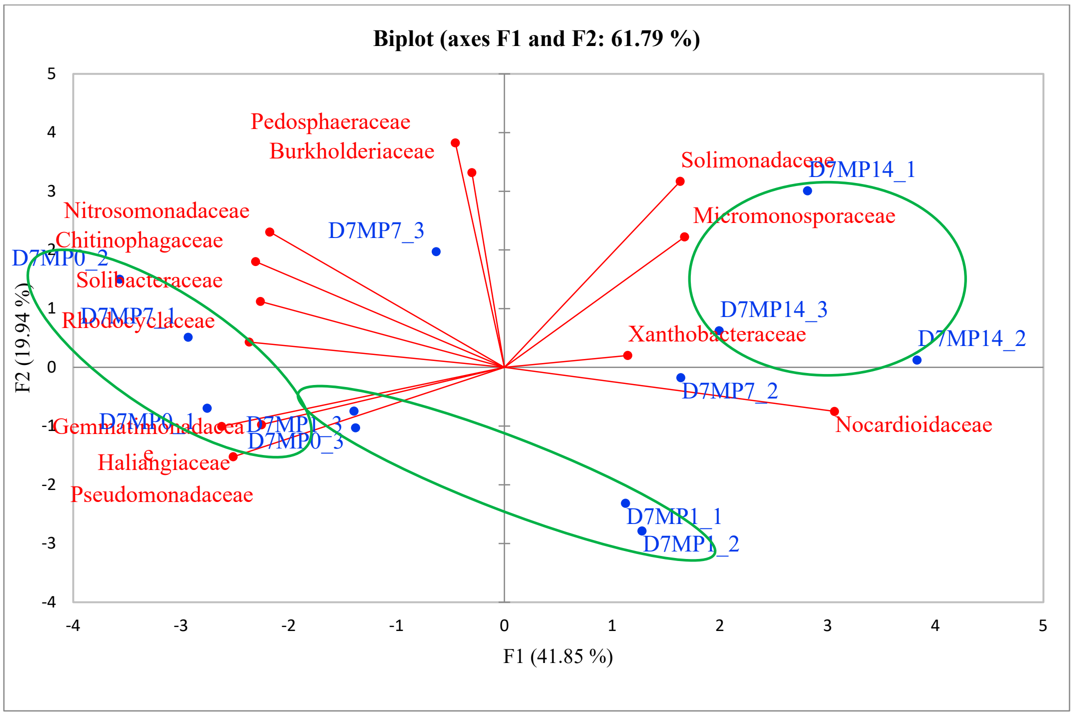

Figure S3. PCA of families at day 7 on PE MPs treatments based on Pearson correlation matrix

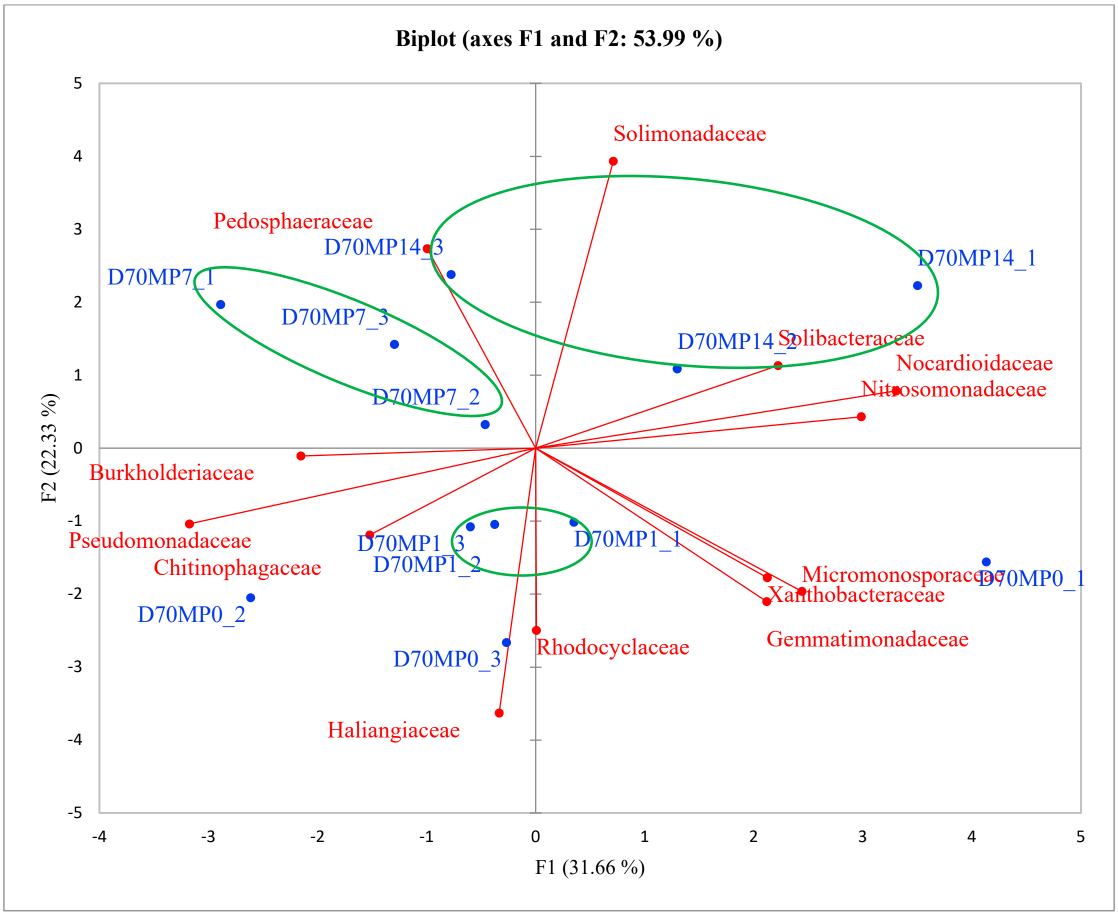

Figure S4. PCA of families at day 70 on PE MPs treatments based on Pearson correlation matrix

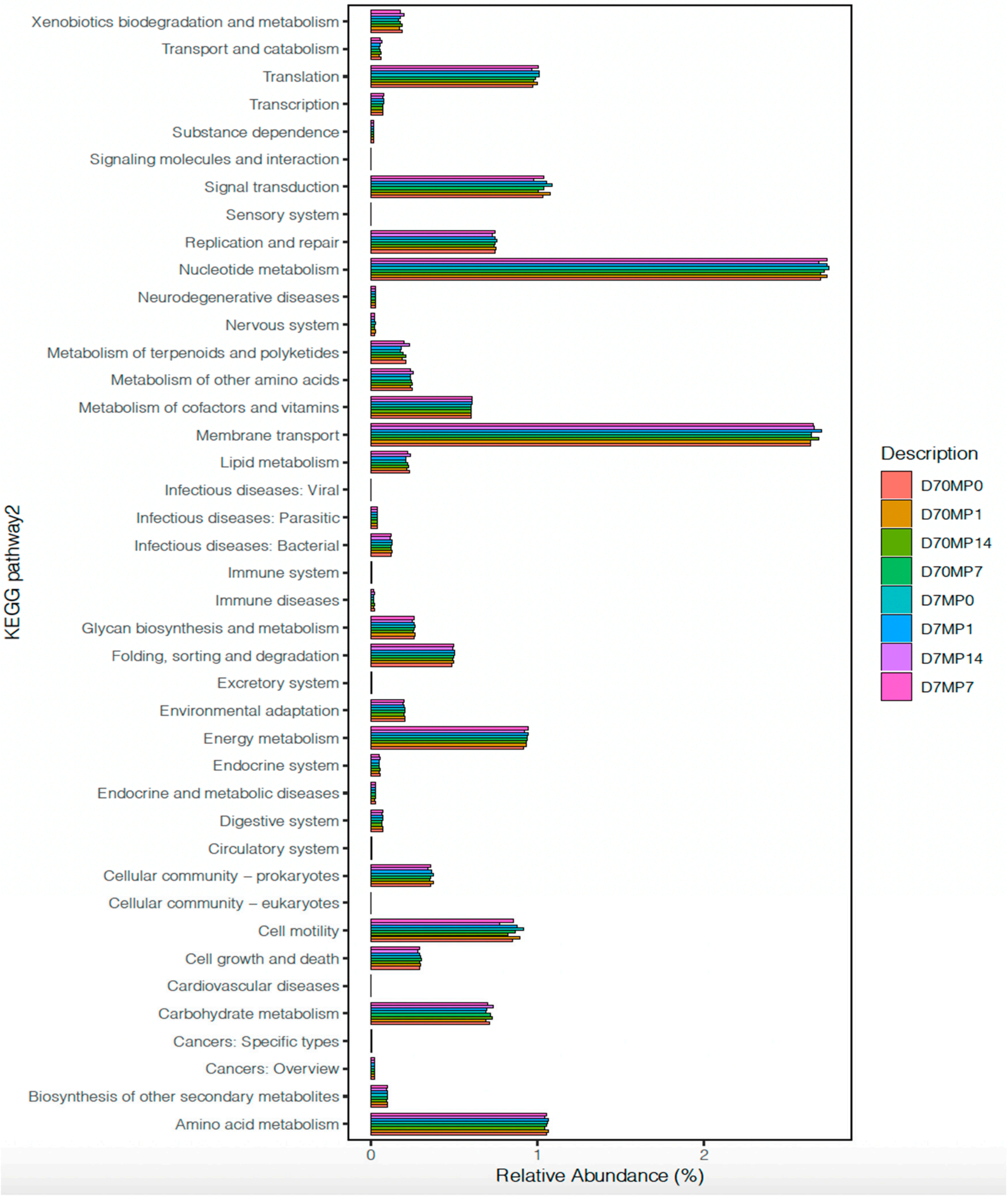

Figure S5. The KEGG pathways by the different PE MPs treatments on day 7 and day 70.

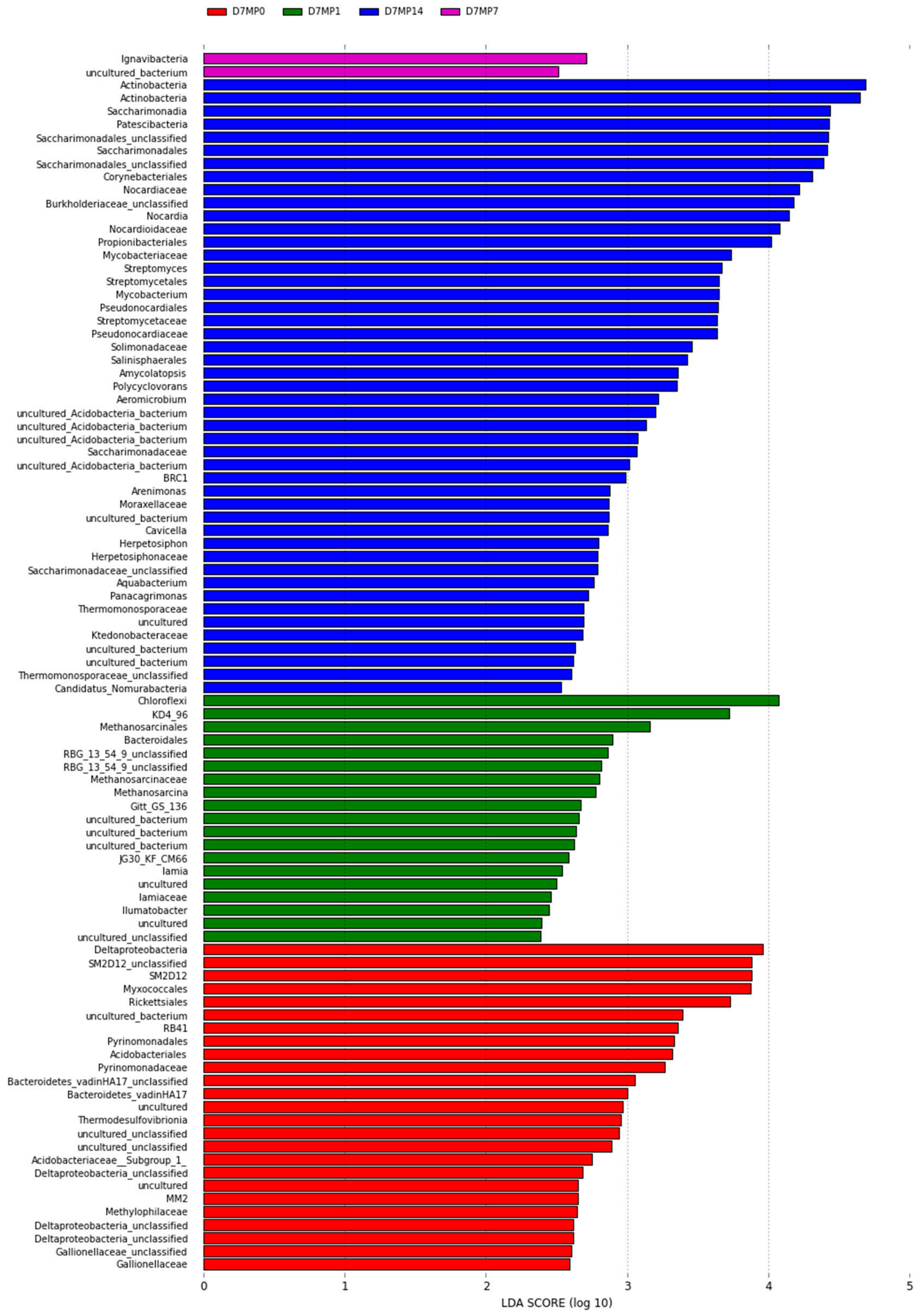

Figure S6. Linear discriminant analysis effect size (LEfSe) from different PE MPs treatments on day 7.

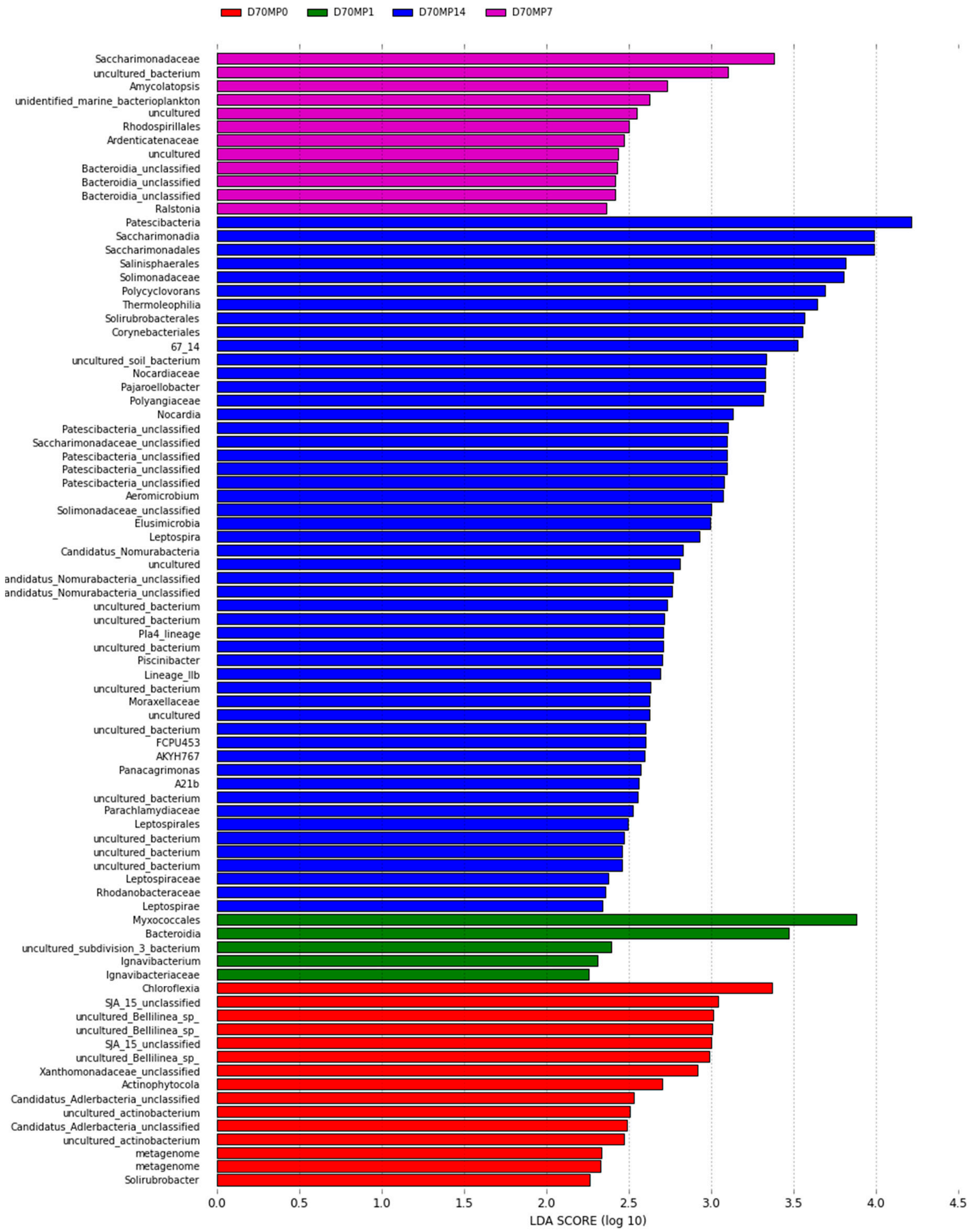

Figure S7. Linear discriminant analysis effect size (LEfSe) from different PE MPs treatments on day 70.

Table S1. Rapid test of pH for PE MPs treatments

	MP_0	MP_1	MP_7	MP_14
Soil with MPs	6.89 ± 0.02 Ad	7.05 ± 0.10 Ac	7.06 ± 0.04 Bb	7.09 ± 0.06 Ba
Water with MPs	6.66 ± 0.27 Bc	6.54 ± 0.13 Bc	9.06 ± 0.60 Ab	10.24 ± 0.14 Aa
Water with washed MPs	6.48 ± 0.02 Bc	6.34 ± 0.03 Cd	6.66 ± 0.11 Bb	6.90 ± 0.23 Ba

Note: Letters a, b, c, and d following the mean ± SD values denote difference of mean at significant level of 0.05 by LSD post hoc test for the row, while letters A, B C, and D for the column.

Table S2. Rapid test of EC (μS/cm) for PE MPs treatments

	MP_0	MP_1	MP_7	MP_14
Soil with MPs	143.53 ± 7.74 Aa	103.07 ± 5.59 Ab	100.32 ± 11.10 Ab	87.20 ± 5.73 Ac
Water with MPs	0.62 ± 0.08 Bc	2.70 ± 0.55 Bc	24.60 ± 2.10 Bb	53.97 ± 3.21 Ba
Water with washed MPs	3.90 ± 0.35 Bc	5.58 ± 0.97 Bc	10.06 ± 2.21 Cb	17.68 ± 3.14 Ca

Note: Footnote resemble that in Table S1

Table S3. AWCD Alpha Diversity at 48 hrs for different PE MPs treatments.

Treatment	H index	Richness	Evenness
MP_0	3.04 ± 0.22 a	13.94 ± 2.12 ab	1.12 ± 0.04 abc
MP_1	3.03 ± 0.21ab	14.56 ± 3.28 ac	1.10 ± 0.05 ab
MP_7	2.96 ± 0.10 b	14.00 ± 2.09 ad	1.13 ± 0.04 ac
MP_14	2.97 ± 0.14 ab	13.11 ± 2.08 ab	1.11 ± 0.04 abc

Note: Letters a, b, c, and d following the mean ± SD values denote difference of mean at significant level of 0.05 by LSD post hoc test for the column

Table S4. Average OTU count on day 7 and 70.

OTU count	MP_0	MP_1	MP_7	MP_14
Day 7	28886	26852	26783	27012
Day 70	29393	25703	25968	25923

Table S5. Influence of PE MPs on bacterial OTU diversity at Day 7 and 70.

	Treatment	H index	Richness	Evenness
Day 7	MP_0	5.00 ± 0.01 a	482.00 ± 16.37 a	0.81 ± 0.01 a
	MP_1	5.02 ± 0.01 ab	485.67 ± 24.50 a	0.81 ±0.00 ab
	MP_7	5.00 ± 0.12 ab	490.00 ± 60.32 a	0.81 ± 0.01ab
	MP_14	4.87 ± 0.02 c	478.00 ± 45.92 a	0.79 ± 0.02 b
Day 70	MP_0	4.96 ± 0.06 b	477.00 ± 27.78 a	0.80 ± 0.02 a
	MP_1	4.98 ± 0.06 b	488.00 ± 42.30 a	0.80 ± 0.01 a
	MP_7	4.95 ± 0.06 b	482.00 ± 13.00 a	0.80 ± 0.01 a
	MP_14	5.12 ± 0.06 a	547.67 ± 67.68 a	0.81 ± 0.01 a

Note: Footnote resemble that in Table S3
